# Association of Chronic Kidney Disease With Small Vessel Disease in Patients With Hypertensive Intracerebral Hemorrhage

**DOI:** 10.3389/fneur.2018.00284

**Published:** 2018-05-02

**Authors:** Yuan-Hsiung Tsai, Meng Lee, Leng-Chieh Lin, Sheng-Wei Chang, Hsu-Huei Weng, Jen-Tsung Yang, Yen-Chu Huang, Ming-Hsueh Lee

**Affiliations:** ^1^Department of Diagnostic Radiology, Chang Gung Memorial Hospital, Chiayi, Taiwan; ^2^College of Medicine, Chang Gung University, Taoyuan, Taiwan; ^3^Department of Neurology, Chang Gung Memorial Hospital, Chiayi, Taiwan; ^4^Department of Emergency Medicine, Chang Gung Memorial Hospital, Chiayi, Taiwan; ^5^Department of Neurosurgery, Chang Gung Memorial Hospital, Chiayi, Taiwan

**Keywords:** chronic kidney disease, hypertension, brain hemorrhages, small vessel disease, magnetic resonance imaging

## Abstract

**Background:**

Chronic kidney disease (CKD) has been closely associated with hypertension and stroke. Although studies have reported the relationship between CKD and cerebral small vessel disease (SVD), the link between CKD, hypertension, and SVD is uncertain. The aim of this study was to investigate the association between CKD and SVD in patients with strictly hypertensive intracerebral hemorrhage (ICH).

**Methods:**

142 patients with acute hypertensive ICH were enrolled in this study. Magnetic resonance imaging was performed to assess imaging markers for SVD. Patients were categorized into three CKD groups based on the degree of kidney dysfunction [glomerular filtration rate (GFR) in milliliters per minute per 1.73 m^2^]: normal kidney function (GFR ≥ 90), mild kidney disease (60 ≤ GFR < 90), and moderate to severe kidney disease (GFR < 60).

**Results:**

The prevalence rate of mild and moderate to severe CKD was 50 and 14.8%, respectively. The stage of CKD was associated with history of chronic hypertension (*p* = 0.046) as well as the prevalence rate of overall and deep cerebral microbleed (CMB) (*p* = 0.001 and *p* = 0.002, respectively). The stage of CKD was a significant risk factor for deep white matter hyperintensity (WMH) (OR 1.848; 95% CI 1.022–3.343, *p* = 0.042), overall CMB (OR 2.628; 95% CI 1.462–4.724, *p* = 0.001), lobar CMB (OR 2.106; 95% CI 1.119–3.963, *p* = 0.021), and deep CMB (OR 2.237; 95% CI 1.263–3.960, *p* = 0.006), even after adjustment for confounders.

**Conclusion:**

In patients with hypertensive ICH, the prevalence of CKD is high even at the early stage of renal function impairment and is associated with the prevalence of CMB and deep WMH. These results reinforce the notion of a link between hypertensive vasculopathy, renal function impairment, and cerebral SVD.

## Introduction

Chronic kidney disease (CKD) is defined by a decreased glomerular filtration rate (GFR) or albuminuria. Evidence has been growing on the interactions between cerebrovascular diseases and impaired renal function. Furthermore, CKD seems to be predictive of several neurological deficits and poor functional outcomes for both ischemic and hemorrhagic stroke (1, 2). The mechanisms of microvasculature damage of brain and kidney are similar. Renal function impairment is characterized by endothelial dysfunction and lipohyalinosis, both of which are similar to the features of cerebral small vessel vasculopathies owing to hypertension, diabetes, and other factors (3, 4). The effect of CKD on incident stroke has been found to be greater in Asians compared with non-Asians (2). This is believed to be related to hypertension, which is generally more common and more severe in Asian than in non-Asian people and is a major risk factor for both CKD and stroke (1, 2, 5).

Primary intracranial hemorrhage (ICH) is responsible for about 15–20% of strokes in Caucasians and with incidence twice as high in the Asians (6). Hypertensive arteriopathy is one of the most common causes of primary ICH and is much more common in Asians, while cerebral amyloid angiopathy (CAA) is the major etiology of lobar ICH (7). A variety of brain small vessels pathology that weakens the vessel wall may lead to vessel rupture and hemorrhage (8). Hypertension drives atherosclerosis into smaller, more distal perforating arteries of the cerebrovascular bed and is the major pathological feature of lacuna infarction (4, 9). In addition, hypertension accelerates lipohyalinosis of the wall of small perforating vessels. Lipohyalinosis, usually in its acute form characterized by fibrinoid necrosis, is the vascular pathology most commonly associated with hypertensive ICH (8). It is prevalent in the basal ganglia of moderate hypertensives and pons in sever hypertensives and is seen not only at the bleeding site but also distant from the hematomas (10, 11). Furthermore, fibrinoid necrosis is associated with aneurysmal dilatation of the penetrating branches of the middle and anterior cerebral arteries, usually the lenticulostriate arteries, which is so-called Charcot-Bouchard aneurysms and were found to co-localize with hypertensive ICH (12–14).

The term cerebral small vessel disease (SVD) describes a range of neuroimaging, pathological and associated clinical features. Features of SVD on neuroimaging include white matter hyperintensity (WMH), enlarged perivascular space (EPVS), cerebral microbleed (CMB), and lacuna (15, 16). The cerebral SVD and CKD have a strong relationship. The kidney and brain share similar and unique susceptibilities to vascular injury since the vasoregulation of the microvasculatures of the two organs are similar (3). Both the glomerular afferent arterioles of the juxtamedullary nephrons and the cerebral small vessels are small, short vessels directly arising from large arteries with high pressure. These kinds of vessels have been referred as “strain vessels,” which also include coronary and retinal arteries (3). Strain vessels have to maintain a strong vascular tone to provide a large pressure gradient in a short distance and are first and severely damaged in hypertensive vasculopathy. Several lines of evidence link SVD to CKD (17–19) and ICH (20–22). However, the imaging markers of SVD as well as subtype of ICH included in each study were heterogeneous. The objectives of this study were to evaluate the prevalence of CKD in patients with strictly deep hypertensive ICH and to investigate the association between hypertension, SVD, and CKD.

## Materials and Methods

### Patients

We performed a study of magnetic resonance imaging (MRI) in 142 consecutive patients hospitalized with acute spontaneous hypertensive ICH. Patients were enrolled after ICH located in the basal ganglia, thalamus, pons, or cerebellum was confirmed by computed tomography. Patients with lobar ICH or hematomas located in atypical location of hypertensive ICH, contraindications to MRI (such as patients who had metallic implants or metallic foreign bodies), irritable that needs heavy conscious sedation and those who refused to consent to participate in this study were excluded. The study was part of an integrated stroke project at Chang Gung Memorial Hospital and was approved by the Institutional Review Board of Chang Gung Memorial Hospital. All patients or their families gave their written informed consent prior to participation in the study.

### MRI Acquisition and Analysis

All data were collected using a 3 T Siemens Verio MRI system (Siemens Medical System, Erlangen, Germany) with a 32-channel head coil. All patients were scanned within 1 month after ICH (mean: 3.9 days; range: 1–14 days). Standard sequences included axial T2*-weighted gradient echo images [repetition time (TR)/echo time (TE) = 600/17.8 ms, excitations = 1, flip angle = 20°, section thickness = 4.0 mm with a gap = 1.2 mm, and matrix size = 256 × 146]; axial fluid-attenuated inversion recovery (FLAIR) images [(TR)/(TE) = 9,000/85 ms, inversion time = 2,500 ms, matrix size 165 × 256, using the same section thickness], and axial T2-weighted (T2WI) turbo spin-echo images [(TR)/(TE) = 3,000/86 ms, matrix size 194 × 256, using the same section thickness].

**Figure 1:**
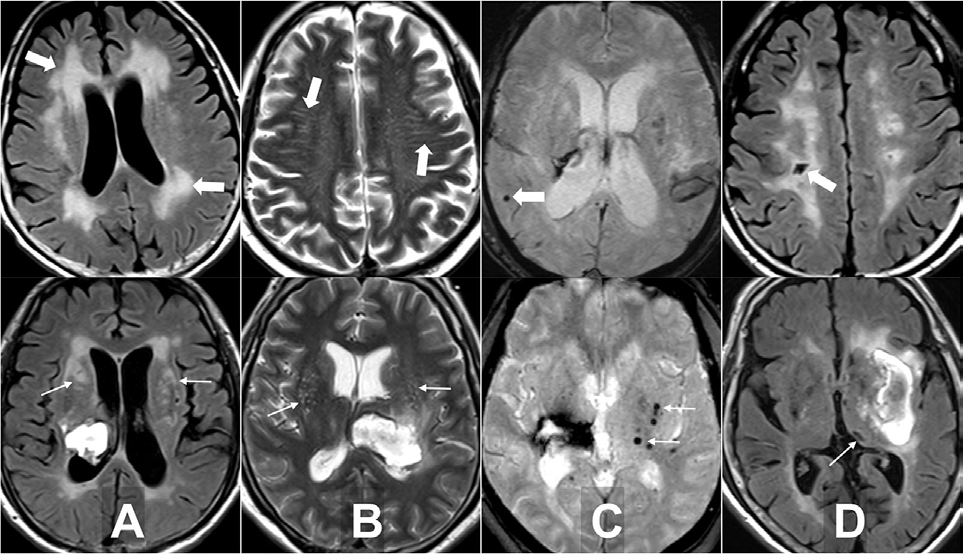
Examples of the four magnetic resonance imaging markers of small vessel disease. **(A)** White matter hyperintensity is a signal abnormality in the white matter that shows hyperintensity on fluid-attenuated inversion recovery (FLAIR) and without cavitation, it is categorized into periventricular (thick arrows) and deep (arrows). **(B)** Enlarged perivascular space is a fluid-filled space that follows the typical course of a vessel and is hyperintensity on T2WI. It is categorized into centrum semiovale (thick arrows) and basal ganglia (arrows). **(C)** Cerebral microbleed is a small, generally between 2 and 10 mm round lesion of signal void with associated blooming seen on T2*-weighted gradient echo images and is categorized into lobar (thick arrow) and deep (arrows). **(D)** Lacuna is a round or ovoid fluid-filled cavity on FLAIR of between 3 and 15 mm in diameter, consistent with previous small subcortical infarct and is categorized into lobar (thick arrow) and deep (arrow).

Four MRI markers of SVD, including WMH, EPVS, lacuna, and CMB, have been previously described (15, 23, 24). In brief, WMH is a signal abnormality of variable size in the white matter that shows hyperintensity on FLAIR and without cavitation. WMH is further categorized into periventricular and deep (basal ganglion and brain stem regions) according to the locations. EPVS is a fluid-filled space that follows the typical course of a vessel as it goes through gray or white matter. EPVS has signal intensity similar to CSF and is hyperintensity on T2WI. EPVS is further categorized into centrum semiovale where it appears linear and basal ganglia where it is round or ovoid with a diameter smaller than 3 mm. Lacuna is a round or ovoid fluid-filled cavity on FLAIR of between 3 and 15 mm in diameter, consistent with previous small subcortical infarct or hemorrhage in the territory of one perforating artery. Lacuna is categorized into lobar and deep (brain stem, deep gray matter, and cerebellum) according to the locations. CMB is a small, generally between 2 and 10 mm round lesion of signal void with associated blooming seen on T2*-weighted gradient echo images. CMB is categorized into lobar (cortical) and deep (brain stem, deep gray matter, and cerebellum) locations (Figure 1). All neuroimaging was reviewed by two neuroradiologists (Y-HT and S-WC) who were blinded to clinical information. In cases of a discrepant score between the two readers, images were reviewed and a consensus was established.

### Clinical Assessments

Demographic data (age and gender), presence of vascular risk factors (hypertension, diabetes, smoking, alcoholism, and previous ischemic stroke or transient ischemic attack), blood pressure [initial blood pressure measured at the emergency room (ER)], previous medications (antihypertensive and antiplatelet agents), as well as other laboratory and clinical assessments [creatinine, National Institutes of Health Stroke Scale (NIHSS) scores, and Glasgow Coma Scale (GCS) scores at the ER] were collected for all patients. Patient outcomes, including GCS score at day 7, Barthel index, and modified Rankin Scale (mRS) score at 3 months, were also estimated.

Estimated GFR according to the modification of diet in renal disease study group equation was calculated for each patient (25). We then categorized patients by degree of kidney dysfunction (GFR in milliliters per minute per 1.73 m^2^): normal kidney function (GFR ≥ 90), mild kidney disease (60 ≤ GFR < 90), and moderate to severe kidney disease (GFR < 60).

### Statistical Analysis

All statistical analyses were performed using the Statistical Program for Social Sciences (SPSS) statistical software (version 18, Chicago, IL, USA). Continuous variables were expressed as medium and interquartile range (IQR) and were compared by performing analysis of variance (ANOVA). *Post hoc* multiple comparisons were performed with Scheffe’s method to test the difference between each group. The Kolmogorov–Smirnov method was used for tests of normality and the Kruskal–Wallis one-way ANOVA test was used when the normality assumption of continuous data was not met (creatinine level, GFR, GCS score at initial and 7 day, NIHSS score, hematoma size, admission systolic, and diastolic pressure as well as 3-month BI). Categorical variables were compared using the Pearson χ^2^ test or Fisher’s exact test. Multivariate binary logistic regression models were used to estimate the risk factors [odds ratio (OR) and 95% confidence interval] for SVD in hypertensive ICH patients. The interobserver reliabilities between two neuroradiologists for measuring the SVDs were tested with two-way intraclass correlation coefficients (ICC) with absolute agreement. All statistical tests were two-tailed and a *p* value of <0.05 indicated a significant statistical difference.

## Results

The clinical and demographic details of the patients are presented in Table 1. All of the patients were diagnosed as hypertensive during admission, regardless of the past history. Among the 142 patients, the prevalence rate of mild and moderate to severe kidney disease was 50.0 and 14.8%, respectively. The mean age of mild kidney disease patients was higher than that of normal function patients (*p* = 0.033) (*Post Hoc* Tests with Scheffe Method: significant difference between normal renal function and mild kidney disease group, *p* = 0.036). Clinical history of hypertension, serum creatinine level, and GFR were significantly different between groups (*p* = 0.046; < 0.001, and < 0.001, respectively). The outcome measurements, including GCS at 7 days as well as mRS and BI at 3 months after ICH, were not associated with stage of renal function impairment. The ICC showed high interobserver reliability for measuring overall WMH, EPVS, CMB, and lacuna (average measures ICC = 0.887, 0.838, 0.958, and 0.862, respectively). Among the four MRI markers of SVD on MRI, patients with worse stage of CKD had a higher prevalence rate of overall CMB and deep CMB (*p* = 0.001 and = 0.002, respectively). In order to exclude patients with mixed hypertensive and CAA, the associations between stage of CKD and patients with CMB of mixed location; pure deep location as well as all patients excluding those with lobar CMBs were estimated and the results showed the trends toward associations but did not reach statistical significance (*p* = 0.076, *p* = 0.065, and *p* = 0.052, respectively).

**Table 1:** Baseline characteristics of study participants (*n* = 142).

Baseline characteristics	Normal kidney function [glomerular filtration rate (GFR) ≥ 90] (*n* = 50)	Mild kidney disease (60 ≤ GFR < 90) (*n* = 71)	Moderate to severe kidney disease (GFR < 60) (*n* = 21)	*p*-Value (*X*2/*F*)
Age, years	58.0 (24.0)	60.0 (17.0)	66.0 (20.0)	0.033*
Male gender	25 (50.0%)	46 (64.8%)	18 (85.7%)	0.015*

**Previous history**				
Hypertension	43 (86.0%)	68 (95.8%)	21 (100.0%)	0.046*
Diabetes	11 (22.0%)	13 (18.3%)	7 (33.3%)	0.342
Smoking	15 (30.0%)	27 (38.0%)	8 (38.1%)	0.632
Alcoholism	14 (28.0%)	18 (25.4%)	6 (28.6%)	0.930
Ischemic stroke or TIA	3 (6.0%)	10 (14.1%)	2 (9.5%)	0.357
Antihypertensive medication	18 (36.0%)	25 (35.2%)	9 (42.9%)	0.810
Antiplatelet medication	1 (2.0%)	3 (4.2%)	2 (9.5%)	0.355

**Initial clinical and laboratory data**				
Creatinine (mg/dl)	0.6 (0.3)	0.9 (0.2)	1.7 (0.8)	<0.001*
Glomerular filtration rate (ml/min per 1.73 m^2^)	104.2 (27.0)	73.2 (15.6)	40.4 (25.5)	<0.001*
NIHSS score	7.5 (12.0)	10.0 (12.0)	10.0 (15.0)	0.622
GCS score	15.0 (4.0)	15.0 (4.0)	15.0 (5.0)	0.825
Size of the hematoma (cm^2^)	8.3 (15.4)	7.8 (10.2)	11.4 (21.3)	0.776

**Admission blood pressure (mmHg)**				
Systolic	191.0 (61.0)	187.0 (44.0)	204.0 (57.0)	0.703
Diastoloic	104.5 (26.0)	108.0.0 (28.0)	105.0 (29.0)	0.515
Mean	130.8 (33.9)	135.0 (25.7)	136.7 (33.5)	0.599

**Outcomes**				
7-day GCS score	15.0 (4.0)	14.0 (4.0)	14.0 (4.0)	0.573
3-month mRS 0–2	26 (52.0%)	28 (39.4%)	10 (47.6%)	0.380
3-month BI	0.0 (14.0)	0.0 (20.0)	0.0 (20.0)	0.744

**Magnetic resonance imaging markers of small vessel disease**				
WMH, overall	34 (68.0%)	54 (76.1%)	18 (85.7%)	0.272
WMH, periventricular	34 (68.0%)	52 (73.2%)	17 (81.0%)	0.527
WMH, deep	19 (38.0%)	39 (54.9%)	13 (61.9%)	0.093
EPVS, overall	46 (92.0%)	61 (85.9%)	18 (85.7%)	0.561
EPVS, centrum semiovale	29 (58.0%)	51 (71.8%)	16 (76.2%)	0.183
EPVS, basal ganglia	44 (88.0%)	61 (85.9%)	18 (85.7%)	0.938
CMB, overall	15 (30.0%)	38 (53.5%)	16 (76.2%)	0.001*
CMB, lobar	10 (20.0%)	18 (25.4%)	10 (47.6%)	0.052
CMB, deep	13 (26.0%)	36 (50.7%)	14 (66.7%)	0.002*
CMB, mixed location	7 (14.0%)	15 (21.1%)	8 (38.1%)	0.076
CMB, pure deep	6 (12.0%)	21 (30.0%)	6 (28.6%)	0.065
All patients excluding lobar CMB	40 (80%)	53 (74.6%)	11 (52.4%)	0.052
Lacune, overall	14 (28.0%)	19 (26.8%)	7 (33.3%)	0.841
Lacune, lobar	8 (16.0%)	13 (18.3%)	5 (23.8%)	0.740
Lacune, deep	4 (8.0%)	7 (9.9%)	2 (9.5%)	0.939

*p < 0.05.

*TIA, transient ischemic attack; NIHSS, NIH Stroke Scale; GCS, Glasgow Coma Scale; mRS, modified Rankin Scale; BI, Barthel Index; WMH, white matter hyperintensity; EPVS, enlarged perivascular spaces; CMB, cerebral microbleed. Continuous variables were expressed as medium and interquartile range and were compared by performing analysis of variance (ANOVA). The Kolmogorov–Smirnov method was used for tests of normality and the Kruskal–Wallis one-way ANOVA test was used when the normality assumption of continuous data was not met. Categorical variables were compared using the Pearson χ^2^ test or Fisher’s exact test*.

To estimate the risk of CKD on SVD, multivariable binary logistic regression analysis was performed to exclude the influence of confounding risk factors including age, male gender, history of diabetes mellitus, and hypertension (Table 2). Age was a significant risk factor for overall WMH (OR 1.060; 95% CI 1.024–1.098, *p* = 0.001), periventricular WMH (OR 1.052; 95% CI 1.018–1.088, *p* = 0.002), deep WMH (OR 1.059; 95% CI 1.026–1.092, *p* < 0.001), overall EPVS (OR 1.044; 95% CI 1.001–1.088, *p* < 0.044), centrum semiovale EPVS (OR 1.055; 95% CI 1.022–1.088, *p* = 0.001), and basal ganglia EPVS (OR 1.049; 95% CI 1.008–1.092, *p* = 0.018). Male gender was a significant risk factor for overall WMH (OR 3.457; 95% CI 1.241–9.630, *p* = 0.018), periventricular WMH (OR 3.847; 95% CI 1.419–10.426, *p* = 0.008), and deep WMH (OR 2.746; 95% CI 1.213–6.218, *p* = 0.015). The stage of CKD was a significant risk factor for deep WMH (OR 1.848; 95% CI 1.022–3.343, *p* = 0.042), overall CMB (OR 2.628; 95% CI 1.462–4.724, *p* = 0.001), lobar CMB (OR 2.106; 95% CI 1.119–3.963, *p* = 0.021), and deep CMB (OR 2.237; 95% CI 1.263–3.960, *p* = 0.006).

**Table 2:** Multivariable binary logistic regression analysis of risk factors for small vessel disease.

	Age		Male		Diabetes mellitus		Hypertension		CKD	
	OR	*p-*Value	OR	*p-*Value	OR	*p-*Value	OR	*p-*Value	OR	*p-*Value
WMH, overall	1.060 (1.024–1.098)	0.001	3.457 (1.241–9.630)	0.018	1.373 (0.489–3.853)	n.s.	1.186 (0.262–5.367)	n.s.	1.695 (0.869–3.303)	n.s.
WMH, periventricular	1.052 (1.018–1.088)	0.002	3.847 (1.419–10.426)	0.008	1.148 (0.419–3.146)	n.s.	1.179 (0.264–5.268)	n.s.	1.480 (0.781–2.806)	n.s.
WMH, deep	1.059 (1.026–1.092)	<0.001	2.746 (1.213–6.219)	0.015	0.787 (0.317–1.950)	n.s.	0.989 (0.208–4.702)	n.s.	1.848 (1.022–3.343)	0.042
EPVS, overall	1.044 (1.001–1.088)	0.044	2.543 (0.643–10.052)	n.s.	0.909 (0.226–3.659)	n.s.		n.s.	0.771 (0.346–1.717)	n.s.
EPVS, centrum semiovale	1.055 (1.022–1.088)	0.001	1.026 (0.449–2.343)	n.s.	0.730 (0.280–1.899)	n.s.	1.352 (0.329–5.559)	n.s.	1.411 (0.777–2.565)	n.s.
EPVS, basal ganglia	1.049 (1.008–1.092)	0.018	2.125 (0.611–7.394)	n.s.	0.789 (0.199–3.128)	n.s.		n.s.	0.938 (0.433–2.032)	n.s.
CMB, overall	1.027 (0.998–1.058)	0.071	1.247 (0.572–2.718)	n.s.	0.806 (0.337–1.927)	n.s.	0.913 (0.204–4.087)	n.s.	2.628 (1.462–4.724)	0.001
CMB, lobar	1.024 (0.991–1.057)	n.s.	1.953 (0.838–4.555)	n.s.	1.656 (0.618–4.442)	n.s.	1.369 (0.242–7.741)	n.s.	2.106 (1.119–3.963)	0.021
CMB, deep	1.023 (0.994–1.053)	n.s.	0.948 (0.437–2.056)	n.s.	0.858 (0.362–2.037)	n.s.	0.545 (0.103–2.876)	n.s.	2.237 (1.263–3.960)	0.006
Lacune, overall	1.011 (0.981–1.043)	n.s.	0.819 (0.360–1.866)	n.s.	0.648 (0.272–1.543)	n.s.	1.978 (0.483–8.091)	n.s.	1.063 (0.590–1.913)	n.s.
Lacune, lobar	1.012 (0.976–1.049)	n.s.	1.066 (0.415–2.742)	n.s.	0.744 (0.275–2.011)	n.s.	1.430 (0.259–7.903)	n.s.	1.258 (0.637–2.482)	n.s.
Lacune, deep	0.996 (0.952–1.043)	n.s.	0.617 (0.160–2.382)	n.s.	0.507 (0.137–1.873)	n.s.		n.s.	0.916 (0.374–2.244)	n.s.

*CKD, chronic kidney disease; WMH, white matter hyperintensity; EPVS, enlarged perivascular spaces; CMB, cerebral microbleed*.

## Discussion

Our study confirms the findings of previous studies that reported a high incidence of CKD in patients with primary ICH. We also showed that CKD was significantly associated with a higher presence of CMB, especially deep CMB in patients with hypertensive ICH. CKD was significantly associated with both lobar and deep CMB as well as deep WMH even after adjustment for confounders.

The prevalence rate of CKD in ICH patients with GFR below 60 ml/min per 1.73 m^2^ was 14.8% in this study, which is similar to the previous reported prevalence rates, which ranged from 11 to 46% (1, 21, 26–29). Interestingly, studies from white populations, such as those published by Yahalom et al. (26) and Molshatzki et al. (27), showed a higher prevalence rate of CKD in ICH patients (35.8 and 46.1%, respectively). By contrast, the prevalence rate of CKD was lower in studies based mainly on Asian populations, such as the SAMURAI ICH study (29) and INTERACT2 study (28) (18.2 and 10.7%, respectively) as well as our result. The reason for this discrepancy may be due to the fact that the etiology of ICH in Asian populations was mostly hypertensive arteriopathy instead of CAA (the major cause of lobar ICH). It has been suggested that Asian people tend to develop hypertension at earlier ages than other races (30) and a longer history of hypertension may cause more profound damage of the end organs and vessels (2). A meta-analysis study by Zhang et al. (31) showed that the risk of stroke associated with hypertension is consistently and significantly greater in Chinese than in whites.

Our results showed that half of the patients had mild CKD (60 ≤ GFR < 90) and the stage of CKD was associated with past history of hypertension. A plausible explanation for these observations may include a suggestion that in Asian people, hypertensive vasculopathy develops at an earlier age, thus leading to long-term atherosclerosis with lipohyalinosis and fibrinoid necrosis of both renal and cerebral arteries. Another explanation is co-existing hypertension accelerates injuries of the cerebral arteries and leads to ICH, even in patients with early CKD. This may be also true in patients of the black race since the association between CKD and the presence and number of CMB in black patients has been reported to be greater than in non-Hispanic whites (21).

Several studies have demonstrated the association of CKD with CMB in different cohorts (21, 32–34). Our results were in line with these reports with a high incidence rate of CMB (76.2%) in moderate to severe CKD patients with ICH, and the stage of CKD being significantly associated with the presence of CMB. We further evaluated the association of CKD with CMB in different brain locations. Compared to lobar CMB, in deep locations CMB was more strongly correlated with stage of CKD, which is possibly a reflection of underlying hypertensive vasculopathy in the cohort. Renal function impairment has been reported to be closely associated with the prevalence of deep or infratentorial CMB, but not pure lobar CMB in hypertensive patients (35, 36). Notably, in our study, the prevalence of overall and deep CMB in ICH patients with mild kidney disease (60 ≤ GFR < 90) was relatively high (53.5 and 50.7%, respectively) as compared to patients with normal kidney function. On the contrary, the prevalence rate of lobar CMB in patients with mild kidney disease (25.4%) was similar to that of patients with normal kidney function but increased rapidly in patients with moderate to severe kidney disease (47.6%). The stage of CKD had trends to be associated with CMB in mixed lobar and deep locations as well as CMB in pure lobar location but did not reach statistical significance. The explanation for these observations may include that hypertensive vasculopathy and associated early renal function impairment may affect small deep perforating arteries and induce deep CMB. But with persistent long-term hypertension and the decline of renal function, the cortical and leptomeningeal arteries will eventually be damaged. This is in line with a recent observation by Pasi et al. (37) that CMB with mixed deep and lobar locations were associated with vascular risk factors similar to hypertensive ICH and severe hypertension could be responsible for ICH or CMB in the lobar location that are generally thought to be associated with CAA. This should be even more true in patients with severe hypertension, poor-controlled diabetes, or end-stage CKD.

In this study, the prevalence of MRI markers of SVD other than CMB, such as WMH and EPVS, was increased with age, and the stage of CKD was only associated with deep WMH. WMH have been reported to be associated with CKD in neurologically normal subjects (17, 19, 38, 39) as well as in ischemic stroke patients (18, 40). However, up to now, the association between CKD and WMH in patients with hypertensive ICH has only been discussed on a limited basis in the literature. In a study of 285 hypertensive subjects without ICH by Umemura et al. (35), microalbuminuria was associated with the prevalence of deep or infratentorial CMB but not advanced WMH. A study by Laible et al. (41) of ICH patients showed similar results that CKD is associated with deep CMB but not WMH. In contrast to these studies with negative results, Ovbiagele et al. (21) reported a significant difference in the grade of WMH between patients with and without CKD. Another study by the INTERACT2 Investigators even showed that WMH grade was associated with death or major disability at 90 days after ICH (20). This discrepancy may be due to different definitions of the grading system for WMH lesions and heterogeneity in patient populations as well as ICH pathophysiology. In a recent study by Charidimou et al. (22), deep WMH was more common in ICH patients with hypertensive vasculopathy compared to those with CAA. Presence of subcortical WMH was associated with lobar CMB while deep CMB were associated with deep WMH. In our study of a strictly hypertensive ICH cohort, the stage of CKD was significantly associated with deep WMH, and the presence of deep WMH had a trend toward increasing in patients with more severe renal function impairment. We infer that chronic hypertension and associated CKD will affect small perforating end-arteries of the deep gray nuclei and deep white matter. Demyelination, gliosis, and axonal loss around perivascular spaces result in deep WMH (42), which represents vascular injury and is a precursor of hemorrhage.

This study had several limitations. First, we included patients with predominantly mild to moderate severity of ICH, and patients with contraindications for MRI, major comorbidities, or requirement for emergent surgical intervention were excluded, thus resulting in selection bias. Second, we excluded patients with lobar ICH but included patients with lobar CMB. This might affect the unity of the cohort for strict hypertensive ICH. To account for this confounding factor, we also presented subgroup analysis excluding patients with lobar CMB. Furthermore, we recorded patient’s history of hypertension, blood pressure, and use of antihypertensive medication at the time of enrollment, but did not estimate if the hypertension had been well controlled or not. Thus, the causality and collinearity between renal function impairment, hypertension, and SVD can’t be well evaluated. Further longitudinal studies with large cohort and close monitoring of the renal function, blood pressure, SVD and rate of stroke or ICH are necessary to clarify this point.

In conclusion, the prevalence of CKD is high in patients with hypertensive ICH. Even an early stage of renal function impairment is associated with hypertensive ICH. CKD is associated with the prevalence of CMB and deep WMH in patients with hypertensive ICH. Lobar CMB has trends to be associated with the stage of CKD, which may support that severe hypertension or renal functional impairment could be responsible for ICH or CMB in the lobar location that are generally thought to be associated with CAA. These results reinforce the notion of a link between hypertensive vasculopathy, renal function impairment, and the imaging biomarkers of SVD. Further longitudinal studies are warranted to clarify how management strategies, such as control of blood pressure and renal function, will affect the dynamic change of these imaging biomarkers and the risks of ICH.

## Ethics Statement

The study was approved by the Institutional Review Board of Chang Gung Memorial Hospital. All patients or their families gave their written informed consent prior to participation in the study.

## Author Contributions

Y-HT: literature review, manuscript writing, and tables making. M-HL, J-TY, and L-CL: clinical data collection, cases review. S-WC and H-HW: imaging analysis. ML and Y-CH: final manuscript review and editing.

## Conflict of Interest Statement

The authors declare that the research was conducted in the absence of any commercial or financial relationships that could be construed as a potential conflict of interest. The reviewer MP and handling Editor declared their shared affiliation.
